# A formal concept analysis and semantic query expansion cooperation to refine health outcomes of interest

**DOI:** 10.1186/1472-6947-15-S1-S8

**Published:** 2015-05-20

**Authors:** Olivier C Curé, Henri Maurer, Nigam H Shah, Paea Le Pendu

**Affiliations:** 1 Université Paris-Est, LIGM - UMR CNRS 8049, 5 bd Descartes, 77454 Marne la Vallée, France; 2 University of Edinburgh,, Edinburgh, Scotland; 3 Stanford University, BMIR Lab, Stanford, Palo Alto, USA

**Keywords:** Health outcome of interest, Ontology, Semantic Query Expansion, Formal Concept Analysis

## Abstract

**Background:**

Electronic Health Records (EHRs) are frequently used by clinicians and researchers to search for, extract, and analyze groups of patients by defining Health Outcome of Interests (HOI). The definition of an HOI is generally considered a complex and time consuming task for health care professionals.

**Methods:**

In our clinical note-based pharmacovigilance research, we often operate upon potentially hundreds of ontologies at once, expand query inputs, and we also increase the search space over clinical text as well as structured data. Such a method implies to specify an initial set of seed concepts, which are based on concept unique identifiers. This paper presents a novel method based on Formal Concept Analysis (FCA) and Semantic Query Expansion (SQE) to assist the end-user in defining their seed queries and in refining the expanded search space that it encompasses.

**Results:**

We evaluate our method over a gold-standard corpus from the 2008 i2b2 Obesity Challenge. This experimentation emphasizes positive results for sensitivity and specificity measures. Our new approach provides better recall with high precision of the obtained results. The most promising aspect of this approach consists in the discovery of positive results not present our Obesity NLP reference set.

**Conclusions:**

Together with a Web graphical user interface, our FCA and SQE cooperation end up being an efficient approach for refining health outcome of interest using plain terms. We consider that this approach can be extended to support other domains such as cohort building tools.

## Background

In applications that use Electronic Health Records (EHRs), such as in drug safety surveillance or observational studies, groups of patients are selected, extracted, compared, and analyzed based on definitions of certain health outcomes of interest (HOIs) [1,2]. Common examples of HOIs include myocardial infarction (MI), chronic obstructive pulmonary disease (COPD), acute renal failure, juvenile idiopathic arthritis, or peripheral artery disease. While the entry points for a search may at times be obvious, their full definitions are anything but easy to obtain. In work that also incorporates clinical text as data inputs, such as discharge summaries or nurses notes, these definitions should also capture variations of terms that are likely to appear in written notes so that the text-mining process has good recall [3]. For example, there are at least 47 distinct ways that we have seen so far for saying 'myocardial infarction' that appears with frequency in real clinical notes. The challenge in defining an HOI emerges due to the complexity and high number of medical and health terminologies. There are currently over 160 terminologies in the Unified Medical Language System (UMLS) Metathesaurus with over 2-million distinct Concept Unique Identifiers (CUIs) and over 6-million distinct strings related to health and medicine. Some of these, like the International Classification of Diseases, Ninth Revision, Clinical Modification (ICD-9-CM), are well-known and often used to define the selection criteria for health outcomes of interest. We have found that most clinicians and researchers will say that choosing from lists of ICD-9 or SNOMED CT codes, is not a good way to perform this task. The problem is that the organization and presentation of concepts in any one of these terminologies, let alone 160 of them, might be intuitive for one person yet completely obtuse to another.

This work addresses these challenges by enabling the user to specify their HOI using plain terms, much like an ordinary search query. We use Semantic Query Expansion (SQE) and Formal Concept Analysis (FCA) [4] to induce a lattice of concepts over hundreds of ontologies and terminologies at once and to find the best-matching concepts. Briefly, a lattice is a partially ordered set where every two elements have a least and greatest upper bounds. FCA is a machine learning technique that uses a lattice to reveal associations between elements of some predefined structure - in this case, a set of ontologies with hierarchies and mappings. SQE leverages the hierarchies and mappings to expand concepts to include all subsumed ones. The system aims for broad coverage initially and asks for feedback on the furthest matches at the highest points of the lattice so that the search space can be rapidly refined with minimal input from the user. At the same time the user is issuing queries and confirming or denying concept matches, we display search results that highlight snippets from clinical notes matching their HOI, which provides instant feedback for the user as they refine their HOI. We have implemented these tools as REST service Web APIs. Used together, FCA and SQE make it possible to order concepts semantically and search for matches that best reflect the intension of the user, *i.e*., the medical concepts she wants to express through a set of proposed terms. Intuitively, the system aims initially for greater coverage and relies on user feedback to improve relevance. The user's search query and feedback essentially helps to pinpoint and prune sections of the lattice until only those concepts that fit their intension remain. The amount of feedback can be minimized by measuring coverage of concepts at each major branch.

### A previous method for myocardial infarction HOI

In [3], a query-driven approach called "2-hop" was used to create a set of HOIs which enabled in the detection of drug-adverse event associations and adverse events associated with drug-drug interactions. Intuitively, the algorithm considers a set of concepts, denoted *C*, and derives all subconcepts *C' *in each ontology *O *available in an ontology repository. A second round of concept derivations is performed over the concepts *C' *to obtain another set of concepts denoted *C"*. Because concepts are mapped across ontologies, the process traverses simultaneously all ontologies that contain *C *(and *C'*), thereby "hopping" across ontologies twice. With this approach, *C" *can capture more concepts from the adjacent ontologies that otherwise would not have been identified with a unique iteration. In theory, a least fixed-point semantics recursion could be used. Nevertheless, recursion does not work well in practice due to differing abstraction levels among ontologies, which induce cycles. After several experimentations, we have found that the "2-hop" approach achieves an adequate balance between soundness and completeness for the current use case.

In fact, that method permitted to recognize events and exposures with sufficient accuracy for a drug safety use case. This accuracy was determined using a gold-standard corpus from the 2008 i2b2 Obesity Challenge. The corpus went through different manual annotations and extensions. That is 16 conditions and their events were annotated in order to evaluate the ability of NLP systems to identify a condition present for a patient given a textual note. Using the set of terms corresponding to the definition of the event of interest and the set of terms recognized by our annotation workflow in the i2b2 notes, we estimated the specificity as well as the the sensitivity of identifying each of the events.

Although efficient, this method requires a significant amount of manual effort, *i.e*., some medical experts have to provide concept identifiers, implying a deep knowledge of some ontologies. The goal of this work is to increase automation by only requiring from the medical experts to provide terms associated to their search. Our goal is also to retain/improve previous results.

### Semantic Query Expansion theory

One of the major assets of ontologies is the set of hierarchical relationships they often include. For every concept, a set of parent or super-concepts will usually induce a directed acyclic graph (DAG) structure for most biomedical ontologies. We can use standard graph traversal algorithms to compute the transitive closure and store the set of all ancestors and descendants of every concept. We should note that ontologies in general can be more structurally complex than a DAG, in which case inference engines should be used to compute the subsumptions hierarchy in place of graph traversal algorithms. SQE is the process of taking a set of concepts as a query and utilizing the transitive closure to expand that set to include all descendants or ancestors depending on whether the goal is to generalize or specialize the query.

### Formal Concept Analysis theory

FCA permits to abstract conceptual descriptions from some objects which are described by some attributes. This process finds practical application in fields such as machine learning, knowledge management, as well as data and text mining. Given a set of ontologies, we represent all objects and their attributes - including hierarchical relations - as a binary matrix. Using the standard machinery of FCA, a concept lattice can be generated from this matrix. Because every lattice is a partial order, FCA will group similar objects according to their attributes in an ordered manner. We utilize these properties to identify improbable relations that are not explicitly stated in the ontology as a means of ranking questions to the user that will cover the greatest benefit in narrowing the SQE search space.

More formally, FCA is based on the notion of a triple K=(G,M,I), where G is a set of objects, M is a set of attributes and I is a binary relation between G and M, i.e., *I *⊆ *G *× *M*. Such a triple is denoted as a *formal context*. Given an object g and an attribute m, (*g, m*) ∈ *I *is interpreted as "object g has attribute m." Moreover, with a formal context, one can define the notion of *formal concepts*, where, for *A *⊆ *G*, we define *A' *= {*m *∈ *M *|∀*g *∈ *A *: (*g, m*) ∈ *I*} and for *B *⊆ *M*, we define *B*' = {*g *∈ *G*|∀*m *∈ *B *: (*g, m*) ∈ *I*}. A formal concept of K is defined as a pair (*A, B*) with *A *⊆ *G, B *⊆ *M, A*' = *B *and *B*' = *A*. A Formal concept hierarchy can be formalized as follows: (*A*_1_, *B*_1_) ≤ (*A*_2_, *B*_2_) ⇔ *A*_1 _⊆ *A*_2 _and *B*_2 _⊆ *B*_1_. The concept lattice of K is the set of all its formal concepts with the partial order ≤.

A formal concept hierarchy follows the set of mathematical axioms that defines a lattice. This is denoted as a *concept lattice *since the relation between the sets of objects and attributes is a Galois connection. Galois connections play an important role in lattice theory, universal algebras, and recently in computer science [5]. Let (*P*, ≼) and (*Q*, ≼) be two partially ordered sets (poset). A Galois connection between *P *and *Q *is a pair of mappings (Φ, Ψ) such that Φ : *P *→ *Q*, Ψ : *Q *→ *P *and: (i) *x *≼ *x*' implies Φ(*x*) ≽ Φ(*x*'), (ii) *y *≼ *y*' implies Ψ(*y*) ≽ Ψ(*y*') and (iii) *x *≼ Ψ(Φ(*x*)) and *y *≼ Ψ(Φ(*y*)), for x,x' ∈ P and y,y' ∈ Q.

## Methods

### Expansion using SQE

The user inputs a free-text query representing their HOI, such as 'pituitary cancer'. The query is tokenized and matched against all synonyms of every concept from our set of ontologies. For UMLS, this results in a set of matching CUIs. This step is purely lexical and does not use any semantics. We utilize the structure of the ontologies to expand the search and identify matches such as 'neoplasm' or 'tumor' that are closely related to the search. We perform this expansion by navigating to all super-concepts in each ontology incrementally until we achieve a minimal cover of lexical matches. Broadening the query using SQE in this way will identify many closely related terms (i.e., increase coverage), but it may also introduce many unrelated ones (i.e., decrease relevance). Thus, we have to prune the expanded set aggressively to improve relevance, which we facilitate using FCA (described in more detail below).

**Figure 1:**
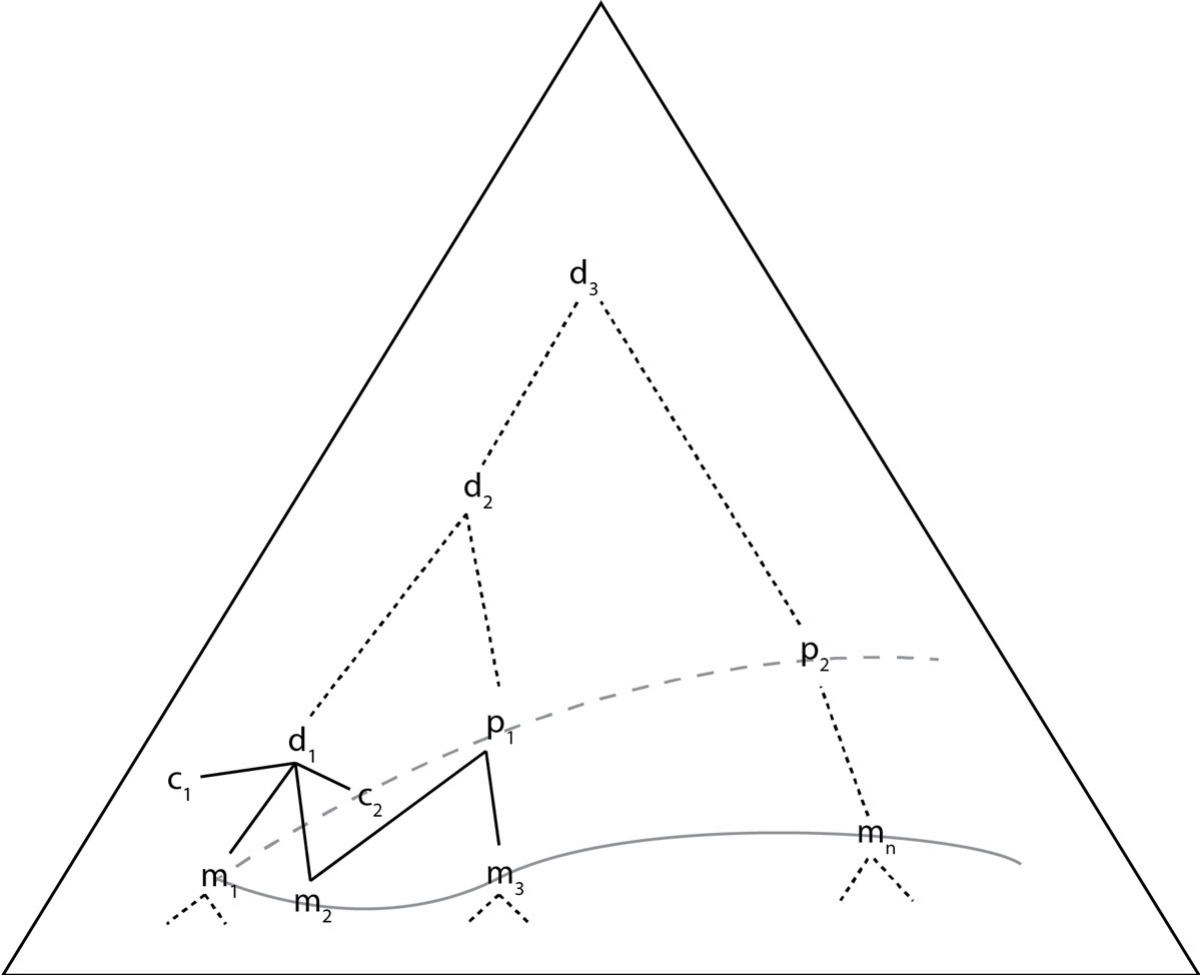
**Overview of ontology concept hierarchy**.

In Figure 1, we provide an abstract representation of the ontology resulting from our methodology where the nodes *m_i_, d_i_, p_i _*and *c_i _*are all ontology concepts and respectively correspond to matching concepts, derived concepts using our method, potential concepts (*i.e*., derived ones that present sufficient qualities to be considered for further processing) and the remaining concepts. For example, the *m *concepts at the bottom represent the initial lexical matches, and the *p *concepts are the set of super-concepts that cover them. Based on the 'poor' coverage of *d*_1 _(perhaps because *c*_1 _and *c*_2 _seem too unrelated to *m*_1 _and *m*_2_) the user may be asked whether *d*_1 _is a fit, and if not, it is pruned (which automatically eliminates *c*_1 _and *c*_2 _and everything else subsumed by *d*_1_) otherwise it would become a potential one, *i.e*., just like *p*_1 _and *p*_2_.

### Pruning using FCA

**Figure 2:**
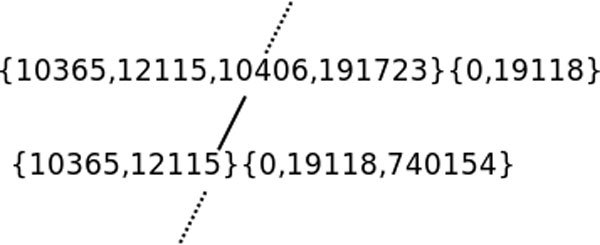
**Extract of Hypertriglyceridemia FCA matrix and lattice**.

Table 1 provides an FCA lattice example using only hierarchical relations for a query on Hypertriglyceridemia. In the lattices we are producing with our approach, both objects (left value at each row) and attributes (top value of each column) are ontology concepts with the attributes corresponding to the super concepts of the objects. This lattice uses internal identifiers: 10365 stands for the Hyperpoproteinemia type IV concept and 0 correspond to the top concept of our set of ontologies. Hence 10365 is a sub-concept of the concepts 0, 19118 and 740154. Intuitively, FCA can identify rectangles such as the one made of columns {0,19118,740154} and rows {10365,12115} or the one made of columns {0,19118}. These rectangles are formal concepts as introduced in Section. The FCA lattice is defined over these rectangles. Figure 2 displays the extract of the lattice corresponding to the table in Table 1.

**Table 1:** Hypertriglyceridemia FCA matrix extract.

	0	19118	740154	6260	**..**.
10365	1	1	1	0	

12115	1	1	1	0	

10406	1	1	0	0	

191723	1	1	0	0	

...					

One advantage of the FCA lattice is to provide a compact representation of the hierarchy that enables us to efficiently find the highest cut among concepts in our ontologies. Using this approach, we are able to obtain the maximal coverage of terms identified via lexical matching. It also enables us to identify concepts worth pruning in the following manner:

Let *F_i _*= 〈*O_i_, A_i_*〉 be a formal concept traversed with *O_i _*and *A_i _*respectively the set of objects and attributes. For each *F_i _*traversed during our top-down navigation of the lattice, we create the two following lists: one denoted $L_{i}^{A}$, corresponding to the transitive closure of the sub-concepts for each element of *A_i _*and another one denoted *L^O^*, corresponding to the transitive closure of the sub-concepts of all *O_i_*. We compute the intersection of $L_{i}^{O}$ with each $L_{i}^{A}$ and we use the ratio $\left|L_{i}^{A}\cap L_{i}^{O}\right|/)\left|L_{i}^{A}\right|$. If this value is below a predefined threshold (denoted pruning threshold), e.g., 75%, then we consider that the considered *A_i _*concept is not relevant to the search, i.e., it has too many sub-concepts not corresponding to sub-concepts of the matched concepts. Otherwise it is relevant and we store it in a candidate list which will be proposed later on to the physician.

**Example (hypercholesterolemia): **Using hypercholesterolemia as an example search query and a set of 18 ontologies, we identify 20 objects and 102 attributes initially, as in Figure 1. This induces a lattice of 67 formal concepts. Its top most formal concept contains all 67 objects with an empty attribute set. The lattice's second level has 2 formal concepts, one with 23 objects (and one attribute) and another one with 17 objects (and one attribute). Both of these concepts have too many sub-concepts not corresponding to the set of sub-concepts of our 20 original objects, hence they are pruned. At the fourth level of the lattice, we discover a first potential concept contained in a formal concept containing 9 objects and 9 attributes one of which has a 75% ratio, i.e., satisfying our pruning threshold. It has 16 sub-concepts out of which only 4 are not covered by the sub-concepts of the 9 objects sub-concepts. Some of these 4 concepts could be unrelated, so we drill down further, identify the specific area of the lattice with the smallest ratio, and ask the user whether this concept is a fit, if not, we prune the lattice above and work at this lower level instead until the user is satisfied. In the example, the labels of the 4 non-covered concepts are: hypercholesterolemia, cholesterolosis, secondary hypercholesterolemia and hyperlipidemia.

### Web-based API

**Figure 3:**
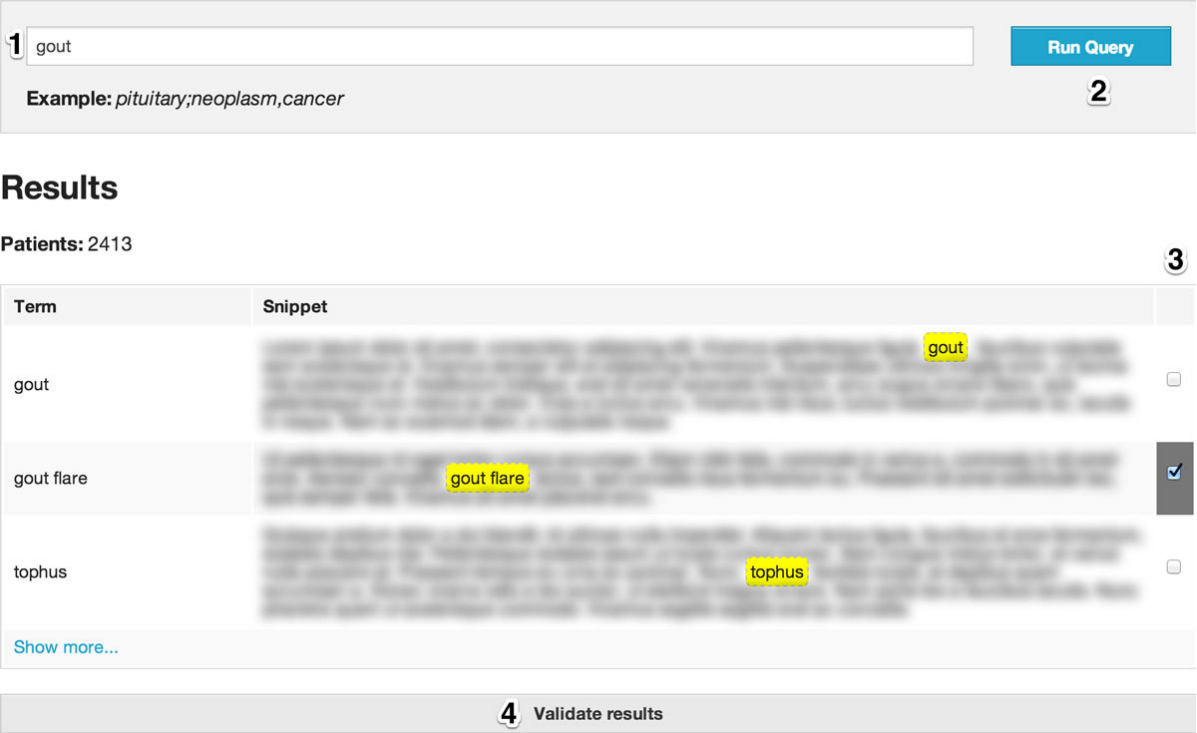
**Example graphical interface based on API**.

Figure 3 presents a web-based interface using the APIs for SEQ and FCA based search and refinement. The user provides and receives feedback in multiple ways (numbered from 1 to 4). First of all, the physician enters some terms associated to her search (area #1) and runs the query (area #2). The terms are sent to REST service APIs, which uses SQE and FCA to identify those concepts that best fit the query, and those that require confirmation from the user. The concepts are also simultaneously matched against a pre-indexed set of clinical notes (area #3), which place those concepts within the context of their real-world use. The user can then confirm or deny the correctness of the match and update the search (area #4). The number of patients is also displayed in area #3, informing the user on how many hits are being discovered.

## Results

In [3], the online Supplementary Data S3 reports that the "2-hop" method identifies HOIs with a sensitivity of 74% and a specificity of 96% for the i2b2 Obesity NLP reference set, henceforth i2b2 obesity. Our evaluation is performed on this same dataset in order to enable a comparison of our method. As such, we consider the set of terms associated to the 16 different conditions surveyed in i2b2 Obesity and retrieve from our database the corresponding concept identifiers. Given this setting, we are able to compute the sensitivity and specificity of our approach. This is presented in Table 2. The experiments have been conducted on commodity hardware running a Java implementation using a MySQL database instance, we thus consider that there are rooms for performance gains.

**Table 2:** i2b2 Obesity NLP reference evaluation.

Condition	Se	Sp	D(s)
Asthma	92.7%	99.1%	3.8

CAD	75.5%	99.4%	5.0

Congestive heart failure (CHF)	74.2%	99.4%	5.5

Depression	69.9%	100%	5.6

Diabetes mellitus	82.0%	99.2%	67.3

Gallstones / Cholcystectomy	81.2%	98.7%	4.9

GERD	56.2%	100%	4.8

Gout	94.4.%	100%	8.7

Hypercholesterolemia	82.4%	100%	4.9

Hypertension	82.6 %	98.6%	8.4

Hypertriglyceridemia	60.0%	98.8%	0.9

Obstructive sleep apnea (OSA)	100 %	100 %	1.5

Osteoarthritis	61.2%	98.9%	2.3

Peripheral vascular disease	66.3 %	99.4%	1.1

Venous insufficiency	77.8%	99.1%	2.8

Obesity	86.1%	99.2%	0.6

**Average**	**77.3%**	**99.1%**	**8.0**

Notes: Se (Sensitivity), Sp (Specificity), D (Duration), CAD (Atherosclerotic cardiovascular disease), GERD (Gastoresophageal reflux disease)

The analysis of our method results is given in 3 dimensions: sensitivity, specificity and processing duration (not including end-user interactions). The obtained statistical measures are encouraging with averages of sensitivity and specificity of respectively 77.3 and 99.1%, hence improving on the results of the less automatized "2-hop" solution. In the first hand, these values have to be considered in the context of a fast processing of potential terms, i.e., duration in seconds range from 0.6 to 67.3. The 2 order of magnitude between the slowest and fastest executions are explained by the size of the matching concepts retrieved from the query patterns, the size of their subsumption relationship transitive closure and the structure of the associated FCA lattice. The slowest, i.e. Diabetes mellitus, involves the computation of matrix of more than 330 objects and 7000 attributes resulting in an FCA lattice of more than 3000 formal concepts out of which most candidates concepts are pruned. In the second hand, the matching and candidate concepts are proposed to the physician for acceptance and rejection. Hence, through an intuitive and user-friendly interface, she is able to easily improve the sensitivity measure. An evaluation on the efficiency and interactivity of the Web interface has yet to be conducted on with physicians on real case scenarios.

Finally, we consider for some of the false negative concepts discovered by our method may end up being positive propositions. Moreover, these propositions come from both the matching (e.g., "Sitosterolemia for hypercholesterolemia" for hypercholesterolemia) and potential (e.g., "h/o: raised blood, familial hyperlipoproteinemia", "fh: raised blood lipids" for hypercholesterolemia while the gold standard contains concepts such as "hyperlipoproteinemia type ii") concepts which confirms the relevance of using a semantic approach. Note that among our true positive, depending on the use case, a significant number of items have been retrieved from the potential concept set, i.e., using our FCA statistical approach.

## Conclusions

We have presented a novel, semi-automatic solution for defining health outcomes of interest. Our work is inspired by previous, manually-intensive work done for the purpose of text-mining clinical notes from EHRs. Our approach is supported by a cooperation between Formal Concept Analysis, to classify, infer and prune discovered concepts, and Semantic Query Expansion, to leverage the hierarchical structure of ontologies. We implemented a RESTful API and a graphical Web-based interface to illustrate the process that users would follow to browse data and refine their HOI query rapidly. First experimentations highlight positive results for the sensitivity and specificity measures. The most promising aspect of this approach consists in the the discovery of positive results not present our i2b2 Obesity NLP reference set. Thus, this approach provides better recall with high precision of the obtained results.

Some of our future goals include (i) running user driven evaluations of the user web interface, (ii) analyzing the acceptance/rejection of physicians in several practical scenarios, (iii) using active learning mechanisms over past query refinements to improve future queries, and (iv) finally studying our method impact's on mining EHRs clinical notes or cohort building tools.

## Abbreviations

API: Application Programming Interface, i2B2: Informatics for Integrating Biology & the Bedside, FCA: Formal Concept Analysis, HOI: Health Outcome of Interest, NLP: Natural Language Processing, SQE: Semantic Query Expansion

## Competing interests

The authors declare that they have no competing interests.

## Authors' contributions

OCC, PLP and NHN drafted the manuscript. OCC and PLP designed the study. PLP and NHN collected data. HM, OCC and PLP were responsible for study conceptualization and implementation of the software. All authors read and approved the final manuscript.
